# Sodium Selenite Acts as an Otoprotectant against Neomycin-Induced Hair Cell Damage in a Zebrafish Model

**DOI:** 10.1371/journal.pone.0151557

**Published:** 2016-03-14

**Authors:** Jiwon Chang, June Choi, Yoon Chan Rah, Myung Hoon Yoo, Kyoung Ho Oh, Gi Jung Im, Seung Hoon Lee, Soon Young Kwon, Hae-Chul Park, Sung Won Chae, Hak Hyun Jung

**Affiliations:** 1 Department of Otorhinolaryngology-Head and Neck Surgery, Hallym University College of Medicine, Seoul, Korea; 2 Department of Otorhinolaryngology-Head and Neck Surgery, Korea University College of Medicine, Seoul, Korea; 3 Laboratory of Neurodevelopmental Genetics, Graduate School of Medicine, Korea University, Seoul, Korea; University of Washington, Institute for Stem Cells and Regenerative Medicine, UNITED STATES

## Abstract

Sodium selenite is a trace element essential for many physiological functions in the body. It is involved in various biological processes; it acts as a cofactor for antioxidant enzymes that protect against free radicals and is reported to limit metal-mediated oxidative DNA damage. In the present study, we investigated the effect of sodium selenite on neomycin ototoxicity in wild-type and transgenic zebrafish (Brn3C: EGFP). Five or six days post-fertilization, zebrafish larvae were co-exposed to 125 μM neomycin and various concentrations (10 μM, 100 μM, 250 μM, and 500 μM) of sodium selenite for 1 h. Hair cells within neuromasts of the supraorbital (SO1 and SO2), otic (O1), and occipital (OC1) lateral lines were analyzed by fluorescence microscopy (n = 10 fish per treatment). Hair cell survival was estimated as the ratio of the hair cell numbers in each group compared to those of the control group that were not exposed to neomycin. Apoptosis and hair cell damage of neuromasts were evaluated using the terminal deoxynucleotidyl transferase (TdT)-mediated dUTP-biotin nick end labeling (TUNEL) assay and 2-[4-(dimethylamino) styryl]-N-ethylpyridinium iodide (DASPEI) assay, respectively. Ultrastructural changes were evaluated using scanning electron microscopy and transmission electron microscopy. Neuromast hair cells were preserved in zebrafish exposed to 125 μM neomycin and 500 μM sodium selenite for 1 h. Sodium selenite protected against neomycin-induced hair cell loss of neuromasts, reduced apoptosis, and prevented zebrafish ultrastructural changes. We propose that sodium selenite protects against neomycin-induced hair cell damage by inhibiting apoptosis, decreasing the disarray of stereocilia, and preventing ultrastructural changes in the neuromast hair cells of the zebrafish.

## Introduction

Ototoxicity refers to medication-induced inner ear dysfunction which results in hearing impairment and/or dizziness [1]. Aminoglycosides are important anti-infection agents used to target gram-negative organisms but were the first ototoxic agents known to cause diverse cochleotoxic and vestibulotoxic effects [2]. Streptomycin and gentamicin are more vestibulotoxic, resulting in dizziness, ataxia, and nystagmus, whereas amikacin and neomycin are more cochleotoxic, resulting in irreversible hearing loss [2]. Neomycin, which was discovered in 1949 [3], was one of the first antibiotics to be developed. Because the systemic administration of neomycin results in cochleotoxicity, the use of this drug is limited to topical application [4]. However, neomycin has been overwhelmingly used in the form of otic drops for treating otitis externa and otitis media. The reported incidence of clinical ototoxicity due to aminoglycoside use ranges from 2% to 5% [5], but since there are lack of well-designed studies regarding ototoxicity from otic drops, and moreover, there are no controlled study evaluating neomycin eardrop risk, the unreported incidence of ototoxicity due to ototopical neomycin cannot be overlooked [6,7].

Selenium is a trace element essential for physiological functions in the brain, liver, heart, and immune system [8]. Low selenium levels are linked to an increase in the mortality of patients who have undergone heart surgery and those with conditions such as sepsis and burns [9–11]. As a component of various selenoproteins, selenium is involved in various biological processes such as antioxidant defense [12], thyroid hormone production [13], and immune responses [14]. Although the exact mechanism by which selenium mediates these physiological processes is unknown, several theories have been proposed, including effects on apoptosis, DNA repair, selenoenzyme formation, carcinogen metabolism, and the immune system [15–17].

The zebrafish is a valuable screening tool for the identification of potentially ototoxic drugs and agents that prevent otoxocity [18]. Zebrafish have hair cells on the surface of their body that function as a part of the lateral line sensory system and it has morphological and functional similarities to mammalian inner ear hair cells. The clusters of 5–20 hair cells, which are similar to the structure of hair cells in mammals, are called a neuromast and the hair cells of neuromasts in the zebrafish lateral line are particularly effective for studying hair cell loss after exposure to ototoxic agents [19–24]. Previous study used 4 or 5 days postfertilization zebrafish and identified that neomycin has ototoxic effect on neuromast of zebrafish [25].

The purpose of the current study was to investigate the effects of sodium selenite on neomycin-induced hair cell damage in a transgenic zebrafish line (Brn3C: EGFP).

## Materials and Methods

### Zebrafish husbandry

The transgenic zebrafish (Brn3C: EGFP) have hair cells, which express green fluorescent protein under a fluorescent microscope without staining [26]. The wild- type and transgenic zebrafish embryos were obtained by paired matings of adult fish maintained at 28.5 ± 1°C in a zebrafish facility at the Korea University Ansan Hospital. Embryos were maintained in an embryo medium (15mM NaCl, 0.5mM KCl, 1mM CaCl_2_, 1mM MgSO_4_, 0.15mM KH_2_PO_4_, 0.05mM NaH_2_PO_4_, and 0.7mM NaHCO_3_) [27], five or six days post-fertilization (dpf) zebrafish larvae were used for the experiment. The present study was approved by the Korea University Institutional Animal Care and Use Committee (No. of Approval: KUIACUC-2012-116). All animal experiments were carried out according to the guidelines of the Animal Care Ethics Committee of Korea University Medical Center and National Institutes of Health (NIH) guidelines.

### Drug administration and evaluation of hair cells in zebrafish

Neomycin powder (C_23_H_46_N_6_O_13_ • 3H_2_SO_4_, CAS-No. 1405-10-3) was purchased from Sigma Chemical Co. (St. Louis, MO, USA). Neomycin solutions were prepared by adding the neomycin powder to the embryo medium. Sodium selenite (Selenase; sodium selenite pentahydrate 1.66 mg/10 ml) was purchased from Biosyn Arzneimittel GmbH (Fellbach, Stuttgart, Germany). We previously reported that neuromasts treated with 125 μM neomycin for 1 h exhibited a viability of 50%, thus a dose of 125 μM neomycin was chosen as an adequate experimental concentration for the current study [28].

**Fig 1:**
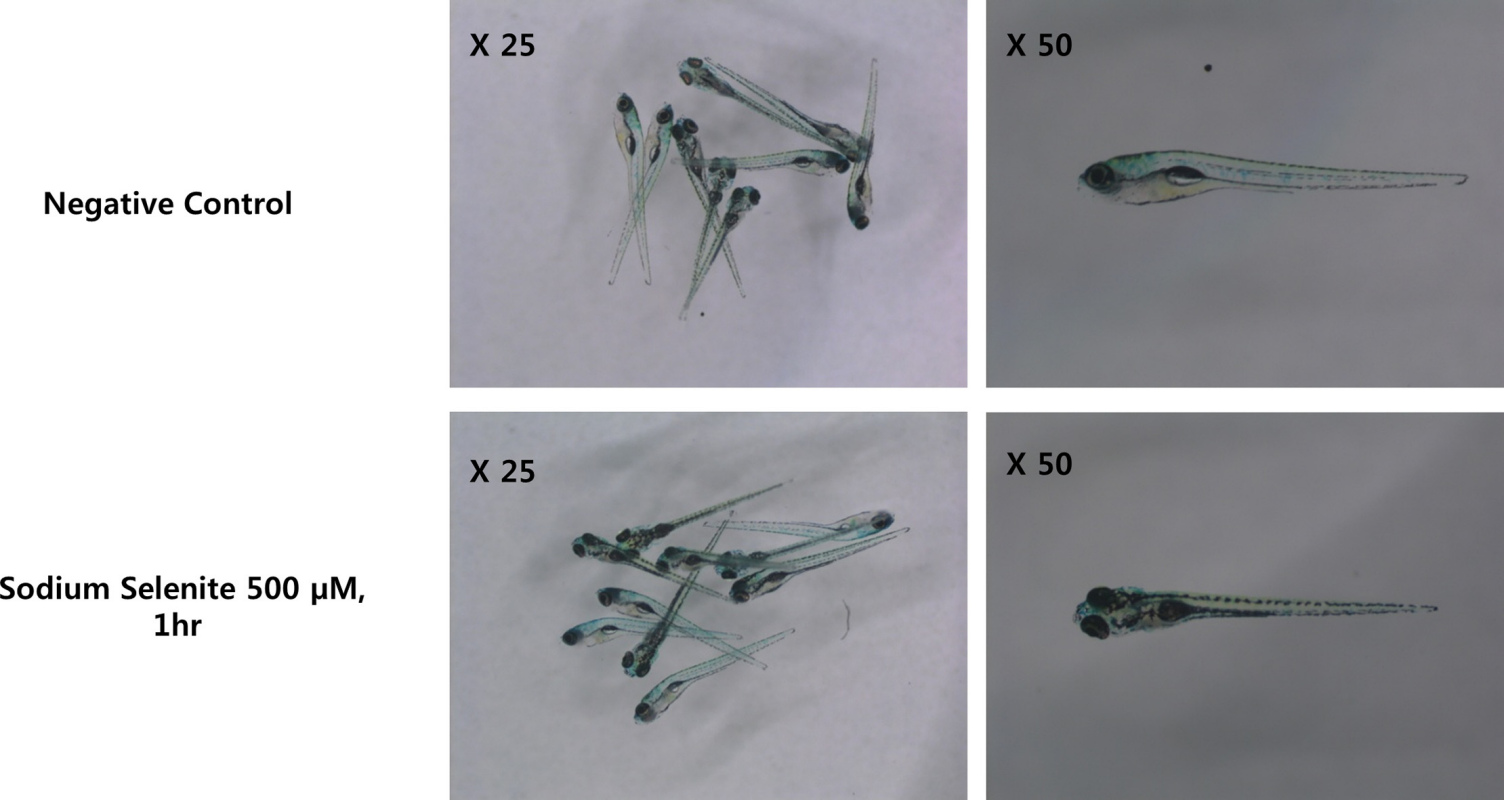
The effect 1hr exposure of 500 μM sodium selenite on zebrafish development. The viability of 6 days post-fertilization zebrafish, three days after the treatment with 500 μM sodium selenite for 1 hr, was evaluated. There were no noticeable developmental differences between zebrafish treated with sodium selenite compare to those of the negative control group.

The five or six days post-fertilization (dpf), zebrafish larvae were, first, exposed the various concentration (10, 100, 250, and 500 μM) of sodium selenite for 1hr. We evaluated daily zebrafish viability and developments in the next three days after sodium selenite exposure for 1 hr (n = 10 fish). All zebrafish were alive and were not observed to have abnormal developments (Fig 1).

Then, five or six days post-fertilization (dpf), zebrafish larvae were exposed to 125 μM concentration of neomycin and various concentrations (10, 100, 250, and 500 μM) of sodium selenite for 1 h, simultaneously (n = 10 fish per treatment). The larvae were then washed with the embryo medium three times. Zebrafish larvae were anesthetized using tricaine (3-aminobenzoic acid 0.4 g/ethyl ester; 100 mL; pH 7, adjusted using Tris buffer) for 5 min as described elsewhere [28, 29]. The zebrafish were mounted using methylcellulose on a depression slide for evaluation under a fluorescence microscope. We previously reported that all experiments were conducted within 2 h because regeneration itself of hair cells in the zebrafish was not significantly observed during that time after chemicals exposure [30].

Hair cells within neuromasts of the supraorbital (SO1 and SO2), otic (O1) and occipital (OC1) were examined [28,29,31]. The total numbers of hair cells of four neuromasts (SO1, SO2, O1, and OC1) were counted in each zebrafish under all experimental and control conditions (n = 10 fish per treatment) by using a fluorescence microscope (LSM5 PASCAL; Carl Zeiss, Germany).

### Evaluation of apoptosis using terminal deoxynucleotidyltransferase (TdT)-mediated dUTP-biotin nick end labeling (TUNEL) assay

The extent of apoptosis in the neuromasts was examined by performing TUNEL staining using an in situ cell detection kit (Roche Molecular Biochemicals, Mannheim, Germany) according to the manufacturer’s protocol. The transgenic zebrafish (Brn3C: EGFP) larvae were exposed to a medium containing 125 μM neomycin and 500 μM sodium selenite for 1 h. Positive control was treated with 125 μM neomycin only for 1h, and negative control was not treated with any drugs. The transgenic zebrafish larvae were then washed with PBS and fixed in 4% paraformaldehyde. Thereafter, they were incubated with 50 μL of TUNEL reaction mixture (TdT and fluorescein–dUTP) at 37°C for 60 min in a humid atmosphere. The TUNEL intensity within four neuromasts (SO1, SO2, O1, and OC1) of the transgenic zebrafish was examined under a fluorescence microscope (AxioCam MRc5, Carl Zeiss) [28,32]. The TUNEL-positive cells were counted per each neuromast and the average number was compared among positive (125 μM neomycin) control group, co-treated group with 125 μM neomycin 500 μM sodium selenite, and negative control group (n = 10 fish per treatment).

### Evaluation of hair cell damage by using 2-[4-(dimethylamino) styryl]-N-ethylpyridinium iodide (DASPEI) assay

The fluorescent dye 2-[4-(dimethylamino) styryl]-*N*-ethylpyridinium iodide (DASPEI; Invitrogen, Carlsbad, CA, USA) was used to stain mitochondria within the hair cells in the wild-type zebrafish. Wild-type fish were used for DASPEI staining experiments due to fluorescence emission overlap with GFP signal in the Brn3c line. It is reported that the hair cells of wild-type zebrafish were similar to that of transgenic zebrafish (Brn3C: EGFP) [22].

The larvae were exposed to a medium containing 125 μM neomycin and 500 μM sodium selenite for 1 h. Positive and negative control was treated as same as above. After the zebrafish larvae were anesthetized using tricaine, the zebrafish larvae were incubated in an embryo medium containing 0.005% DASPEI for 15 min. These larvae were analyzed under a fluorescence microscope and the most clear single plain image was evaluated as described elsewhere [25, 28, 30].

The areas of DASPEI in four neuromasts (SO1, SO2, O1, and OC1) were measured in one side of each fish using Image J (Version 1.48, National Institutes of Health). The average area of DASPEI stained in neuromast were compared among positive (125 μM neomycin) control group, co-treated group with 125 μM neomycin 500 μM sodium selenite, and negative control group (n = 20 fish per treatment).

### Ultrastructural analysis in zebrafish lateral line hair cells

To examine the ultrastructure of the transgenic zebrafish lateral line hair cells by using scanning electron microscopy (SEM), three 5-dpf zebrafish were exposed to 125 μM neomycin and 500 μM sodium selenite for 1 h, prefixed by immersion in 2% glutaraldehyde in 0.1 M PBS, and post-fixed for 2 h in 1% osmic acid dissolved in PBS. The specimens were treated with a graded series of ethanol and *t*-butyl alcohol, dried in a model ES-2030 freeze dryer (Hitachi, Tokyo, Japan), platinum coated using a model IB-5 ion coater (Eiko, IB-5, Japan), and analyzed by field emission-SEM using a model S-4700 microscope (Hitachi) as previously described [30,33].

For examination using transmission electron microscopy (TEM), three 5-dpf zebrafish larvae were exposed to 125 μM neomycin and 500 μM sodium selenite for 1 h, and were fixed for 4 h at 41°C in a mixture of 2% glutaraldehyde and 2% paraformaldehyde in 0.1 M sodium PBS (pH 7.3) immediately after resection. The specimens were post-fixed with 1% Osmium tetraoxide at room temperature in the same buffer for 2 h, dehydrated using a graded series of ethanol, and embedded in epoxy resin. And then, we analyzed a series of semi-thin cross sections (1 μm) that were stained with toluidine blue. Each specimen block was scanned using a light microscope for gross inspection of the neuromasts. Ultra-thin sections (70 nm) were cut and mounted on copper grids, stained with uranyl acetate and lead citrate, and examined using a model H-7600 transmission electron microscope (Hitachi) as described previously [24,28,30,34]. Most evident SEM and TEM images were taken in three 5-dpf zebrafish.

### Statistical analysis

All values are presented as mean ± standard deviation. One-way analysis of variance was used for multiple comparisons. A *post hoc* analysis was performed using Tukey’s honestly significant difference (HSD) test. P < 0.05 was considered statistically significant. Statistical analysis was performed with IBM SPSS 20.0 for Windows (IBM, Armonk, NY, USA)

## Results

### The effect of sodium selenite on hair cell numbers in neuromasts

**Fig 2:**
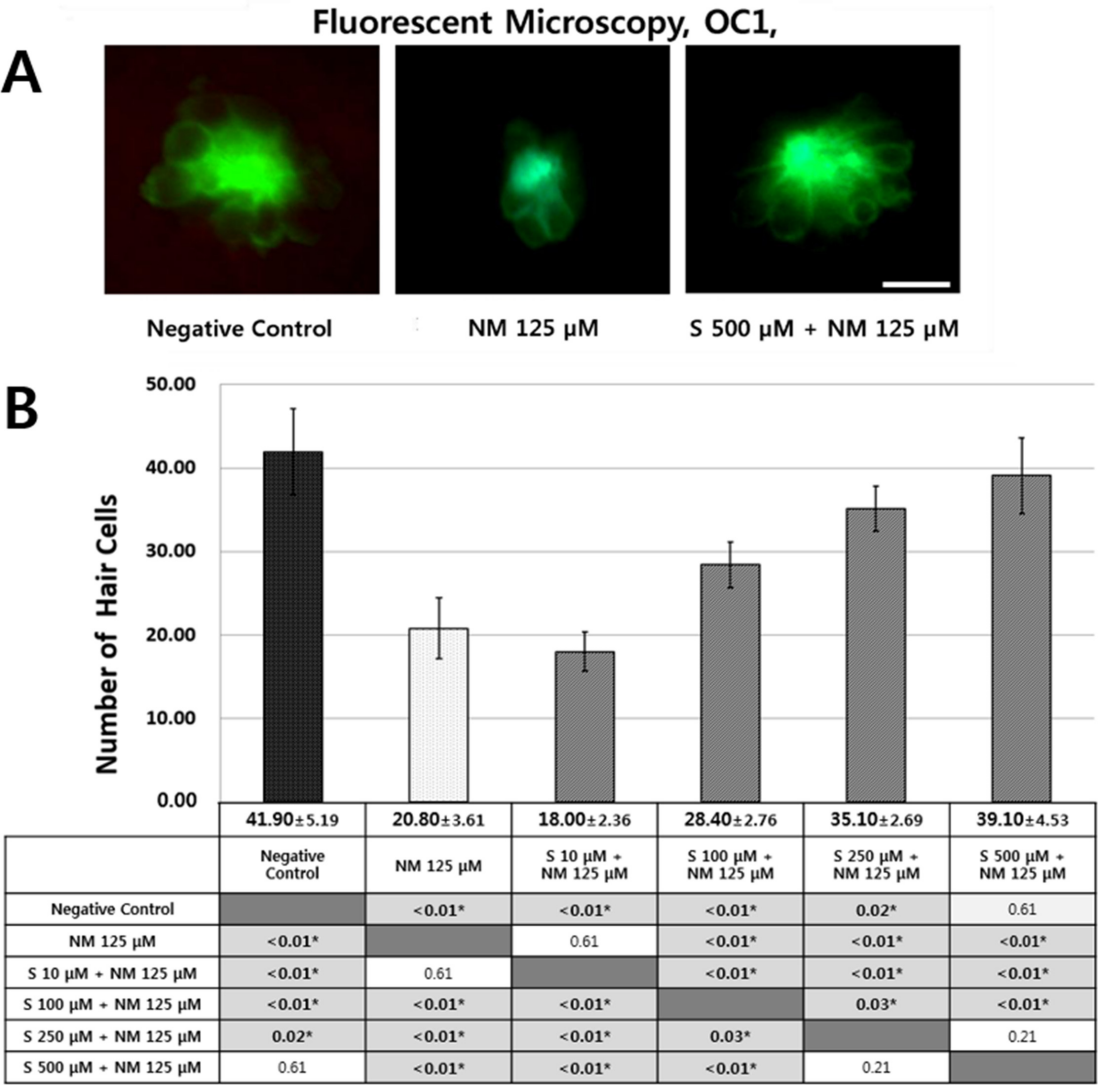
Fluorescent microscopy (OC1, ×40) and quantitative assay for hair cell damage in the five and six days post-fertilization (dpf) transgenic zebrafish (Brn3C: EGFP). The 5 and 6-dpf transgenic zebrafish were treated simultaneously with 125 μM neomycin and various concentrations (10, 100, 250, and 500 μM) of sodium selenite for 1h. Treatment with neomycin resulted in a significant decrease in the number of hair cells, but 500μM sodium selenite most attenuated the hair cell damage (A). Hair cells from four neuromasts (SO1, SO2, O1, and OC1) were analyzed. The total hair cells of negative control group had 41.90 ± 5.19 cells. Treatment of the transgenic zebrafish with 125 μM neomycin for 1 h significantly decreased the number of hair cells in the neuromast (20.80 ± 3.61 cells). Sodium selenite protected against neomycin-induced hair cell loss in a dose dependent manner. The concentration of 500 μM of sodium selenite had significantly protective effect against neomycin (*: statistically significant) (n = 10 fish per treatment, B). NM 125 μM: 20.80 ± 3.61 cells, p<0.01, compared to negative control; S 10 μM + NM 125 μM: 18.00 ± 2.36 cells, p<0.01 and 0.61, compared to negative control and NM 125 μM, respectively; S 100 μM + NM 125 μM: 28.40 ± 2.76 cells, p<0.01, p<0.01, and p<0.01, compared to negative control, NM 125 μM, and S 10 μM + NM 125 μM, respectively; S 250 μM + NM 125 μM: 35.10 ± 2.69 cells, p = 0.02, p<0.01, p<0.01, and 0.03, compared to negative control, NM 125 μM, S 10 μM + NM 125 μM, and S 100 μM + NM 125 μM, respectively; S 500 μM + NM 125 μM: 39.10 ± 4.53 cells, p = 0.61, p<0.01, p<0.01 p<0.01, and p = 0.21, compared to negative control, NM 125 μM, S 10 μM + NM 125 μM, S 100 μM + NM 125 μM, S 250 μM + NM 125 μM, respectively (B). * statistically significant: Scale bar = 10 μm. NM, neomycin; S, sodium selenite.

Transgenic zebrafish (Brn3C: CGFP) has green colored neuromasts that could be visualized under a fluorescent microscope without fluorescent staining. The relationship between each condition and hair cell survival was examined in the lateral line neuromast hair cells. The total number of hair cells per fish was decided from the 4 neuromasts (SO1, SO2, O1, and OC1). The total number of hair cells from four neuromasts were counted to investigate the changes in neuromast numbers after exposure to 125 μM neomycin and various concentrations (10, 100, 250 and 500 μM) of sodium selenite. Treatment of the zebrafish with 125 μM neomycin for 1 h significantly reduced the number of hair cells in the neuromasts (Fig 2A). Sodium selenite reduced neomycin-induced hair cell loss from the neuromasts in the dose-dependent manner. In neomycin-treated zebrafish, the total hair cell count was 20.80 ± 3.61 cells. However, co-exposure 500 μM sodium selenite increased the total hair cell count to 39.10 ± 4.53 (Fig 2B). The sodium selenite concentration of 500 μM was determined to be the most effective in preventing hair cell loss, and therefore, was used in the following studies.

### The effect of sodium selenite on cellular apoptosis

**Fig 3:**
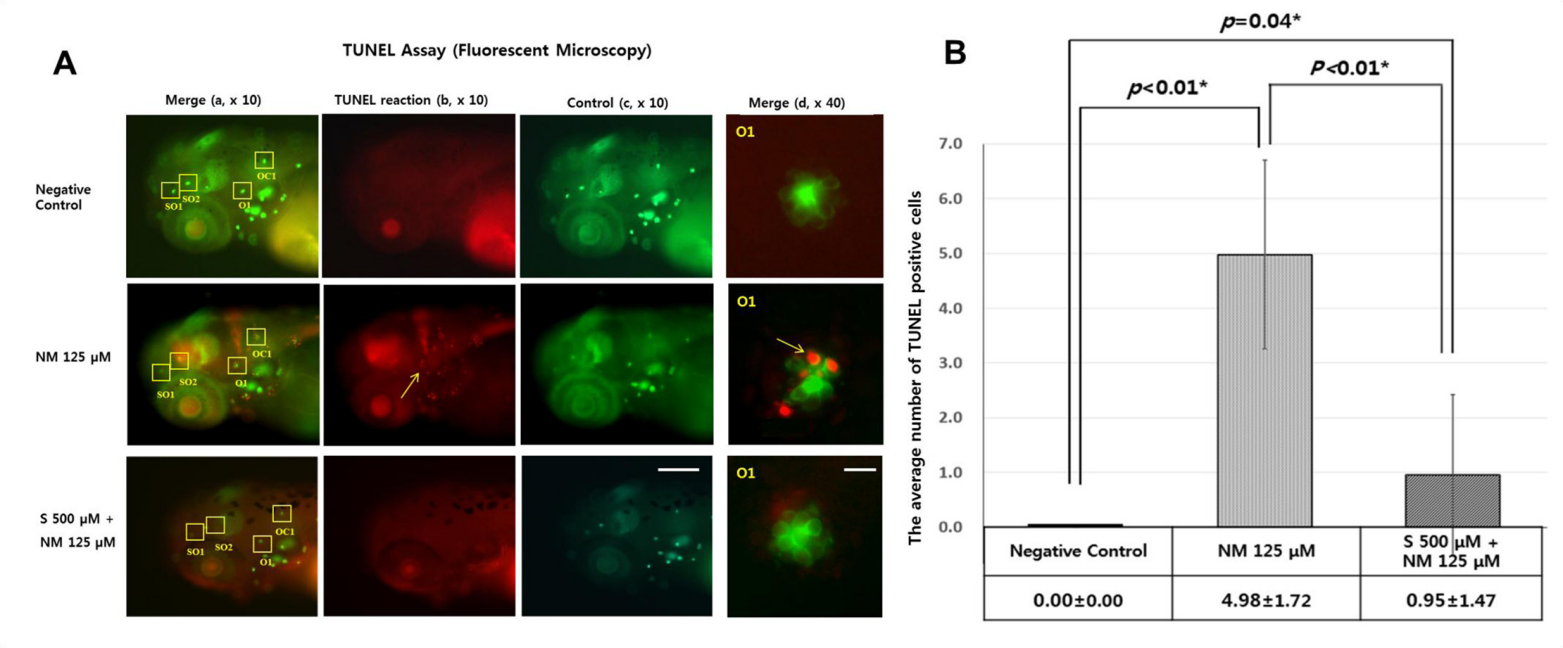
Quantitative analysis of apoptotic cell death by TUNEL assay. TUNEL staining as a quantitative assay was performed to determine the protective effect of sodium selenite against neomycin-induced apoptosis. Apoptotic cells are marked as light red dots in red-colored fish after TUNEL staining as observed under a fluorescent microscope (arrow indicates TUNEL-positive cells in TUNEL reaction (b) column and merge (d) column). Light red dots were not visible in the negative control zebrafish (A). A comparison of the color intensity between the group treated with 125 μM neomycin and the group treated with 500 μM sodium selenite for 1 h showed that the number of TUNEL-positive cells significantly decreased in the 5 and 6-dpf transgenic zebrafish treated with sodium selenite, and that apoptotic cell death of the neuromasts hair cells was attenuated by sodium selenite (green and red-colored fish in the left column, combination of control and TUNEL reaction (a), × 10; red-colored fish in the left middle column, TUNEL reaction (b), × 10; green-colored fish in the right middle column, control for TUNEL reaction (c), × 10; green and red-colored neuromast in the right column, merge for control and TUNEL reaction by high magnification (d), × 40) (A). The concentration of 500 μM of sodium selenite reduced significantly neomycin-induced TUNEL positive cells in four neuromasts (SO1, SO2, O1, and OC1) (*: statistically significant) (B). Negative control: TUNEL positive cells = 0.00 ± 0.00; NM 125 μM: the average TUNEL positive cells, 4.98 ± 1.72; S 500 μM + NM 125 μM: the average TUNEL positive cells = 0.95 ± 1.47; n = 10 fish per treatment, *: statistically significant. (B). Scale bars = 200 μm (c); 20 μm (d). NM, neomycin; S, sodium selenite.

The extent of apoptosis in the 4 neuromasts (SO1, SO2, O1, and OC1) was examined by performing TUNEL staining. TUNEL staining is a quantitative assay to determine the protective effect of sodium selenite on neomycin-induced apoptosis of neuromasts. Exposure of zebrafish to neomycin increased the number of TUNEL-positive apoptotic cells, which are observed as light red dots under the fluorescence microscope. However, sodium selenite treatment decreased the number of TUNEL-positive cells, indicating that apoptosis of hair cells can be inhibited by 500 μM sodium selenite (Fig 3A). In 125 μM neomycin only treated zebrafish, the average number of TUNEL-positive cells within neuromasts was 4.98 ± 1.72 cells. However, co-treatment with 500 μM sodium selenite seemed to be protective for apoptosis with decreased the average number of TUNEL-positive cells down to 0.95 ± 1.47 cells (n = 10 fish per treatment, *p*<0.01) (Fig 3B)

### The effect of sodium selenite on hair cell damage

**Fig 4:**
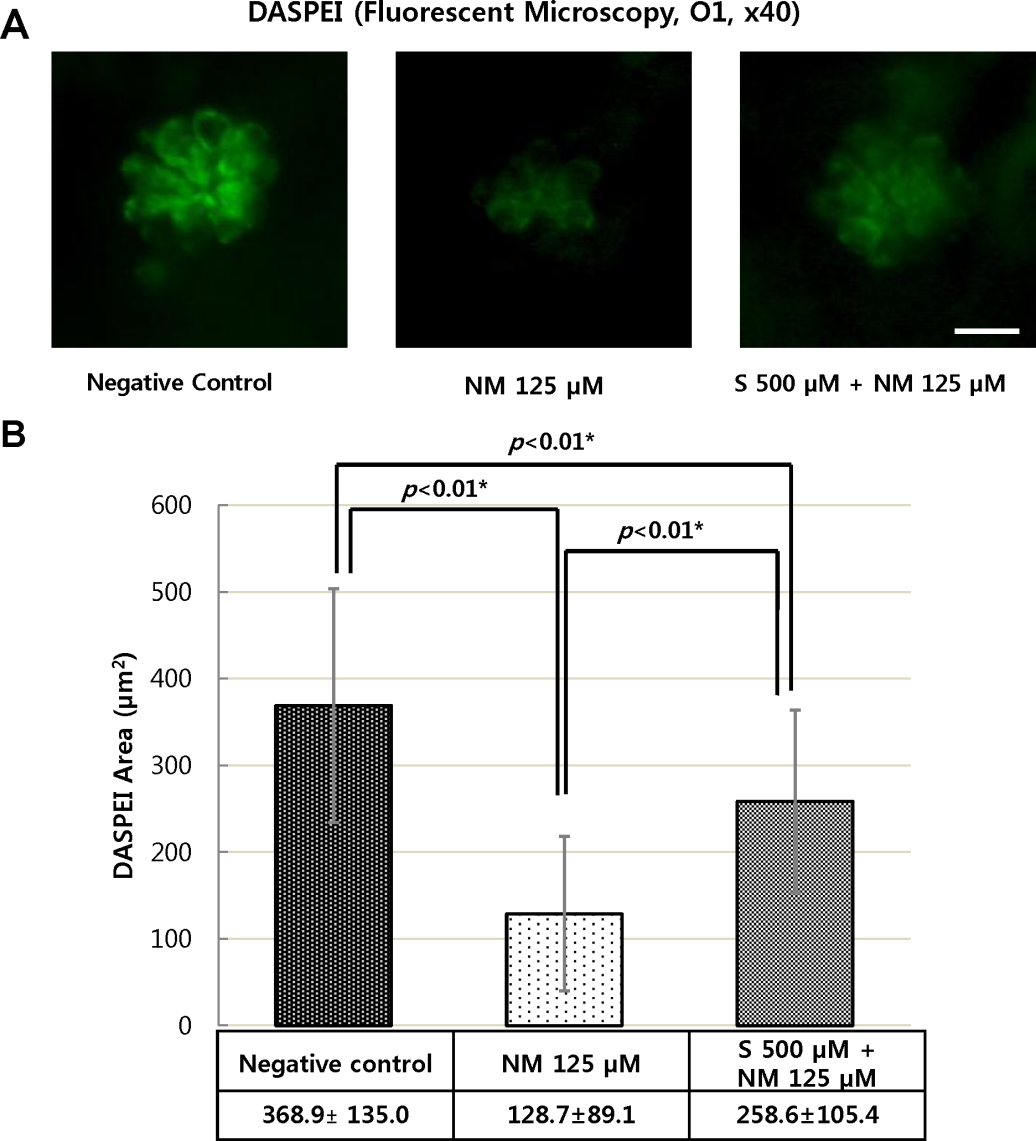
Analysis of hair cell damage by DASPEI assay (×40). The wild type zebrafish were treated with 125 μM neomycin and 500 μM sodium selenite for 1 h. Staining hair cells with DASPEI showed that treatment with 125 μM neomycin caused reduced numbers of hair cells in neuromast. However, the co-treatment with 500 μM sodium selenite protected reducing number of hair cell staining with DASPEI (A). The concentration of 500 μM of sodium selenite preserved significantly the average DASPEI area in four neuromasts (SO1, SO2, O1, and OC1) (n = 20 fish per treatment; *: statistically significant) (B). Negative control: the average DASPEI area = 368.9 ± 135.0 μm^2^; NM 125 μM: the average DASPEI area = 128.7 ± 89.1 μm^2^; S 500 μM + NM 125 μM: the average DASPEI area = 258.6 ± 105.4 μm^2^(B). NM, neomycin; S, sodium selenite. Scale bar = 10 μm.

DASPEI was performed to stain mitochondria and revealed that hair cells of four neuromasts (SO1, SO2, O1, and OC1) maintained their DASPEI area via sodium selenite. Treatment of the zebrafish with 125 μM neomycin increased the amount of cellular damage, as shown by decreased the average DASPEI area, present in hair cells. Conversely, co-treatment of zebrafish with 500 μM sodium selenite protected against the cellular damage (Fig 4A). In 125 μM neomycin only treated zebrafish, the average area of DASPEI within four neuromasts was 128.7 ± 89.1 μm^2^. However, co-exposure 500 μM sodium selenite increased the average DASPEI area to 258.6 ± 105.4 μm^2^ (n = 20 fish per treatment, p<0.01) (Fig 4B).

### Ultrastructural changes in zebrafish lateral line hair cells

**Fig 5:**
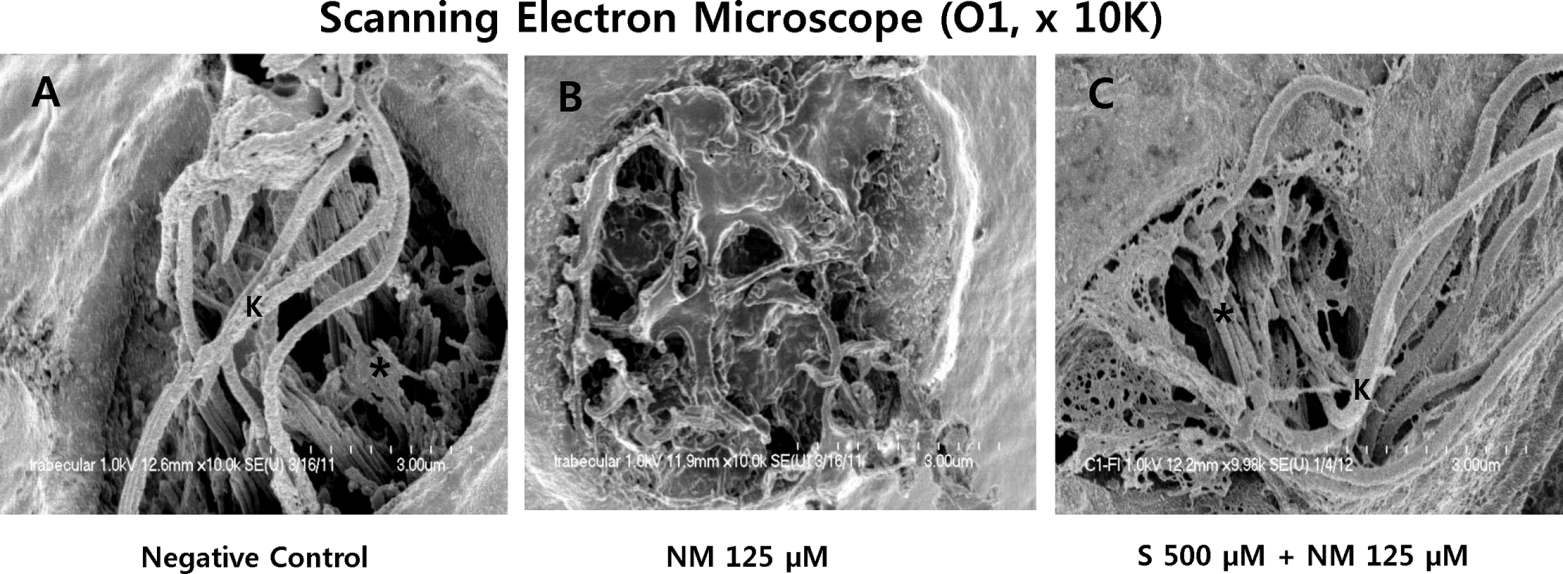
Scanning electron microscopy (SEM, O1, x 10K). The kinocilium (K) and the stereocilia bundles (asterisk) of hair cells in neuromast were clearly visible in the negative control (A). However, when the 5-dpf transgenic zebrafish were treated with 125μM neomycin for 1 h, the kinocilium and the stereocilia bundles were affected and severely disrupted (B). Sodium selenite provided nearly complete protection against neomycin-induced the damage of the kinocilium (K) and the stereocilia bundles (asterisk) in the neuromasts (C). Images were obtained in three 5-dpf zebrafish for each group. Scale bar (at the bottom of each figure, one space) = 3 μm. NM, neomycin; S, sodium selenite.

By using scanning electron microscopy (SEM), we observed that the kinocilium and the stereocilia bundles of hair cells were intact in the control group (Fig 5). When the zebrafish larvae were treated with 125 μM neomycin for 1 h, neuromast hair cells were affected and disrupted. Concurrent treatment with sodium selenite protected against neomycin-induced damage of the kinocilium and the stereocilia bundles in neuromasts.

**Fig 6:**
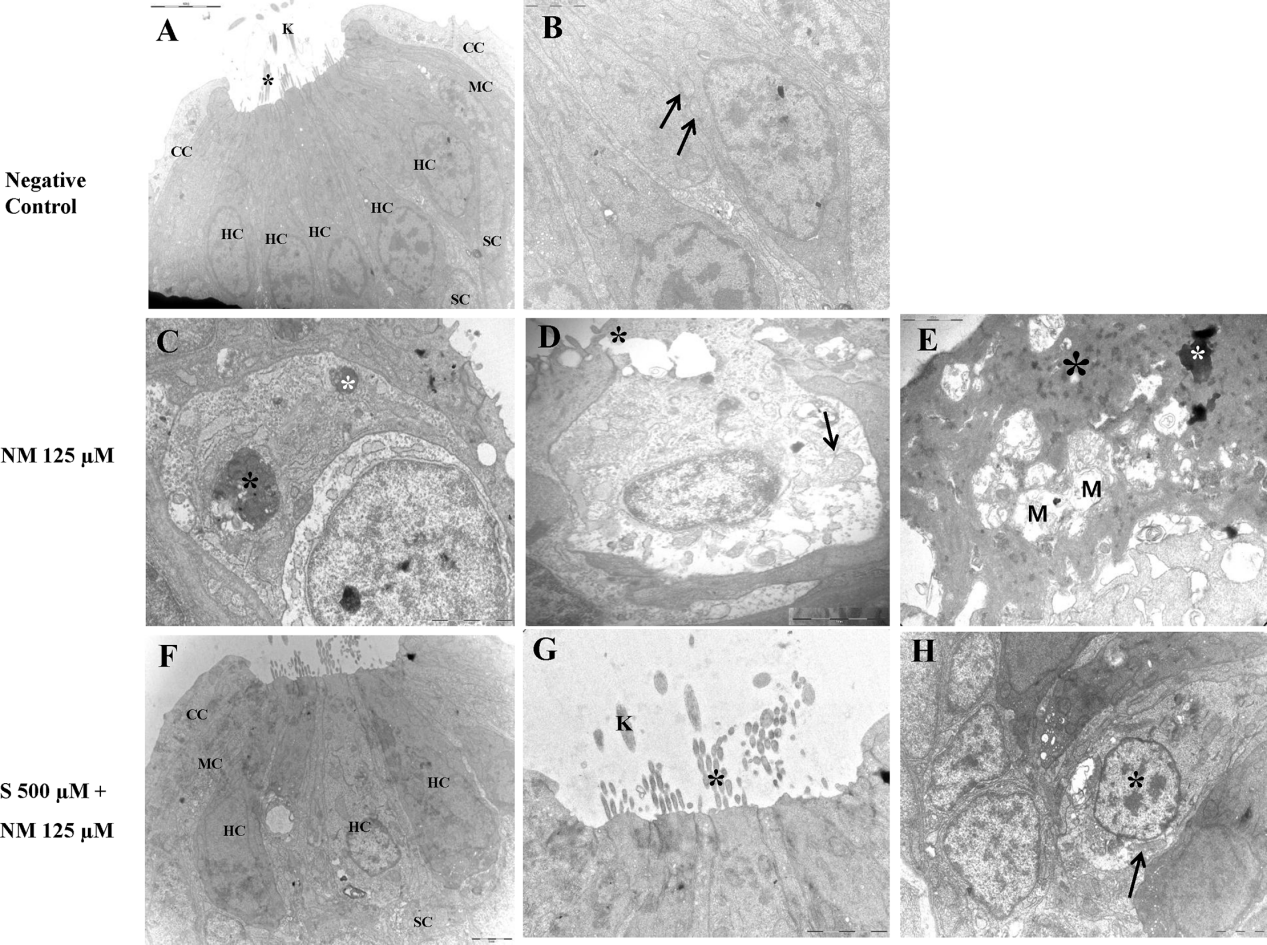
Transmission electron microscopy (TEM). TEM of Normal control (A and B); TEM of zebrafish treated with 125 μM neomycin only (C-E); TEM of zebrafish treated with 125 μM neomycin and 500 μM sodium selenite (F-H). The sterocilia (black asterisk) and the kinocilium (K) from each hair cell are clearly visible (× 8K) (A). A normal-sized mitochondria (arrow, × 25K) (B). The hair cells were severely damaged and showed a condensed nuclei (black asterisk) and pyknotic nuclei (white asterisk) (× 20K) (C). The collapse of the apical surface of neuromasts was typically evident. A hair cell showed extrusion of cytoplasm (black asterisk) and the swollen mitochondria (arrow) (× 20K) (D).Severely damaged hair cells show a severely degenerating cytoplasm (black asterisk), a fragmenting condensed nucleus (white asterisk), and multiple swollen mitochondria (M) (× 30K) (E).When 500 μM sodium selenite was applied, structure of neruomasts were nearly complete protected. Nuclear damage such as condensed cytoplasm and pyknotic nuclei were not showed. (× 5 K) (F). The structure of the stereocilia (black asterisk) and the kinocilium(K) are preserved (× 20 K) (G). The normal-sized nucleus (black asterisk) and mitochondria (arrow) is shown (× 20 K) (H). NM, neomycin; S, sodium selenite; HC, hair cell; SC, supporting cell; CC, crescent cell; MC, mantle cell; BM, basement membrane. Scale bars (at the top or the bottom of each figure, one space) = 5μm (A); 2μm (B); 2μm(C); 1μm (D); 1 μm (E); 2μm (F); 2μm (G); 2μm (H). NM, neomycin; S, sodium selenite. Images were obtained in three 5-dpf zebrafish for each group.

TEM (negative control; Fig 6A and 6B) showed that the treatment of zebrafish larvae with 125 μM neomycin remarkably destroyed kinocilium and stereocilia bundles of hair cells and fused stereocilia. There were cellular changes such the presence of dense cytoplasm and swollen mitochondria, as well as ejection of the cytoplasm and the nucleus (Fig 6C–6E). However, neomycin-induced structural damage was reduced by treatment with sodium selenite (Fig 6F–6H). When sodium selenite was present, the degree of nuclear condensation and number of pyknotic nuclei decreased and the size of the mitochondria was normal (Fig 6F–6H).

## Discussion

In the current study, neomycin treatment increased cellular apoptosis, and enhanced hair cell loss in the lateral line of transgenic zebrafish. Neomycin induced cellular changes in the zebrafish, including increasing the density of the cytoplasm, swelling of mitochondria, and ejection of the cytoplasm and the nucleus. These results are in accordance with those of other studies of zebrafish lateral line changes, which showed swelling of mitochondria, heterogeneous cellular changes, and decreased hair cells within 15–30 minutes of exposure to neomycin [24].

In cases of aminoglycoside ototoxicity, a variety of free-radical species, including both oxygen and nitrogen radicals, are detected in the inner ears [35]. These reactive species are believed to initiate the apoptotic cascade [5] and the mitochondria release apoptogenic factors into the cytoplasm to activate caspases in the intrinsic apoptotic pathway. However, caspases are activated by ligand binding to death receptors such as Fas and TNFR1 in the extrinsic apoptotic pathway [36]. It has been demonstrated that aminoglycosides generate reactive oxygen species (ROS) within hair cells and the ROS subsequently cause either direct or indirect hair cell damage, which ultimately leads to apoptotic cell death and disarray of stereocilia [37, 38]. ROS-mediated hair cell damage is thought to be a result of the affinity of aminoglycosides for iron, and that the aminoglycoside-iron complex enhances iron-catalyzed oxidation and thereby promotes ROS formation [39,40]. Neomycin is an externally used product in clinical settings, and little information is available about its ototoxicity toward inner ear hair cells. However, it is known that the injection of 10^−3^ mol/L neomycin (2 ml) into the unilateral cochlea via the round window membrane induces the loss of all homolateral cochlear and vestibular hair cells and hearing and vestibular function abnormalities in adult C57BL/6 mice [41]. ROS formation by neomycin is reported to play a role in neomycin-induced ototoxicity, and antioxidant compounds is assumed to inhibit intracellular apoptotic pathways triggered by neomycin [42,43]. Our previous reports also demonstrated that neomycin elevated ROS formation and induced apoptosis [44]. The ototoxic effects of neomycin are likely mediated by c-Jun n-terminal kinase (JNK) and caspase-9 activation [45]. It has also been suggested that mitochondrial p53 participates in promoting aminoglycoside-induced hair cell death in zebrafish [46].

Sodium selenite is a trace element essential for many physiological functions, and although the exact role of selenium is unknown, it is believed to be an important mediator of apoptosis, DNA repair, and selenoenzyme formation [15]. The importance of seleniumis that this metal acts as a cofactor of antioxidant enzymes, such as glutathione peroxidase, a protein that protects against free radicals [47]. Other selenium-bound proteins, such as thioredoxin reductase, also ameliorate oxidative stress by reducing disulfide bridges present in other proteins and thus regenerating oxidant-reactive thiol groups [48]. Moreover, selenium dioxide (SeO_2_) and selenite ion (SeO_3_^2-^) can coordinate with Fe (II) and inhibit the amount of iron-mediated oxidative DNA damage [49,50].

Ma *et al*. have reported that long duration (120 hr) exposure of selenite resulted in abnormal development including the absence of neurons in the brain, trunk and abnormal heart function during an embryonic period of zebrafish [51]. However, in the current study, the zebrsfish were exposed the various concentration of sodium selenite for 1hr. We evaluated daily zebrafish viability and developments in order to determine a toxic shock of sodium selenite itself in the next three days after 500 μM sodium selenite exposure for 1 hr (n = 10). And all zebrafish were alive and were not observed abnormal developments. These finding are in contrast to the results of Ma *et al*.

In the present study, co-treatment of sodium selenite reduced neomycin-induced hair cell loss in the neuromasts and decreased apoptosis of hair cells, in a dose-dependent manner. Sodium selenite also preserved the ultrastructure of neuromasts in the lateral line, protected against neomycin-induced damage of the kinocilium and the stereocilia bundles, decreased the degree of nuclear condensation and number of pyknotic nuclei, and maintained mitochondrial size. But although results of present study indicate that sodium selenite reduces neomycin-induced ototoxicity in transgenic zebrafish, further studies are needed to elucidate the mechanism of sodium selenite on inner ear function. Ou *et al*. have demonstrated that quinoline structures, which are known to have *in vitro* antioxidant activities and to protect DNA from harmful chemical reactions, affected the uptake of aminoglycoside into hair cells and subsequently promoted hair cell survival by blocking or limiting neomycin uptake [52, 53].

The limitation of our study is the lack of specific information about the uptake of neomycin. Also, since the area of the neuromast can vary from basal to apical ends, using single plain images of the neuromasts (e.g. Fig 4) instead of z-stack images which describes the state of the whole neuromasts, is another limitation of our study.

## Conclusions

To our knowledge, this is the first study to investigate the protective effect of sodium selenite against neomycin-induced ototoxicity in the neuromasts of zebrafish. The results of the present study show that sodium selenite reduced neomycin-induced apoptosis, decreases the disarray of sterocilia, and prevented ultrastructural changes in the neuromast hair cells of the zebrafish.
